# Supplementary material for: Comparative Thermophysiology of Marine Synechococcus CRD1 Strains Isolated From Different Thermal Niches in Iron-Depleted Areas
Source: Front Microbiol. 2022 May 9;13:893413. doi: 10.3389/fmicb.2022.893413 (PMC9124967; doi:10.3389/fmicb.2022.893413)
Supplement: Supplementary file 1 [file Data_Sheet_1.PDF]

# Ferrieux et al.
# Supplementary Figures

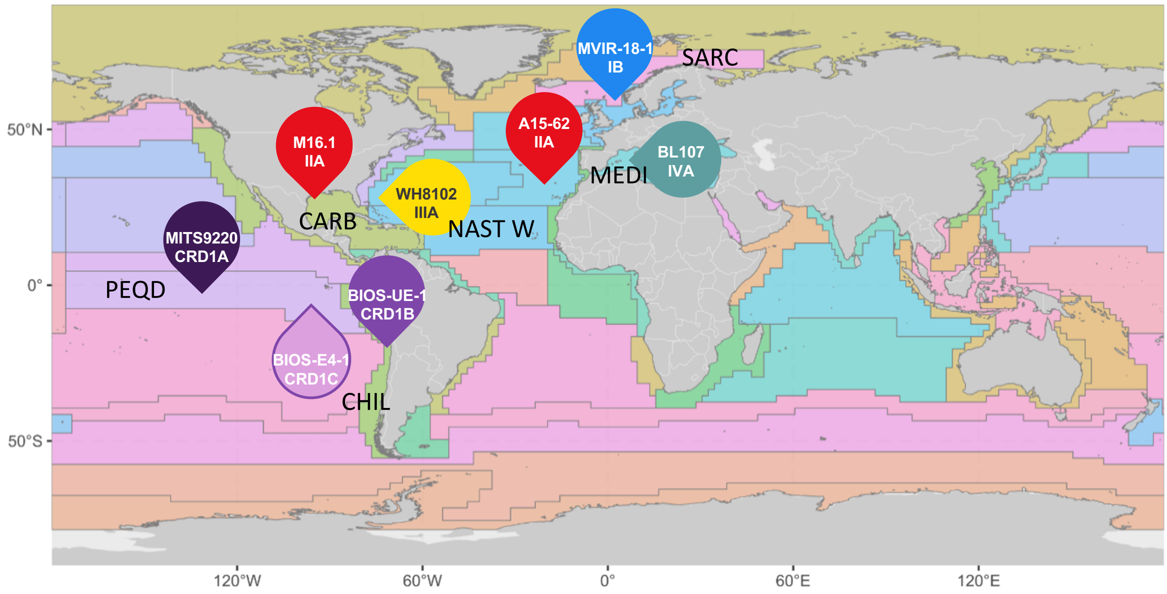


**Supplementary Figure 1. Isolation sites of the *Synechococcus* strains used in this study.** Isolation site of each strain is indicated on the map by a bubble arrow colored according to their corresponding ESTUs indicated below the strain name. Longhurst provinces (Longhurst A., 2007, Ecological Geography of the Sea, Academic Press, London) are shown as a colored background shown in the insert. Only provinces from which at least one strain has been isolated are indicated on the map using the following abbreviations: PEQD (Pacific equatorial divergence, Pacific, Trade wind), CHIL (Chile-Peru, current coastal province, Pacific, Coastal), NAST W (Northwest Atlantic subtropical gyral, Atlantic, Westerly), CARB (Caribbean, Atlantic, Trade wind), MEDI (Mediterranean Sea, Atlantic, Westerly), SARC (Atlantic sub-Arctic, Atlantic, Polar).

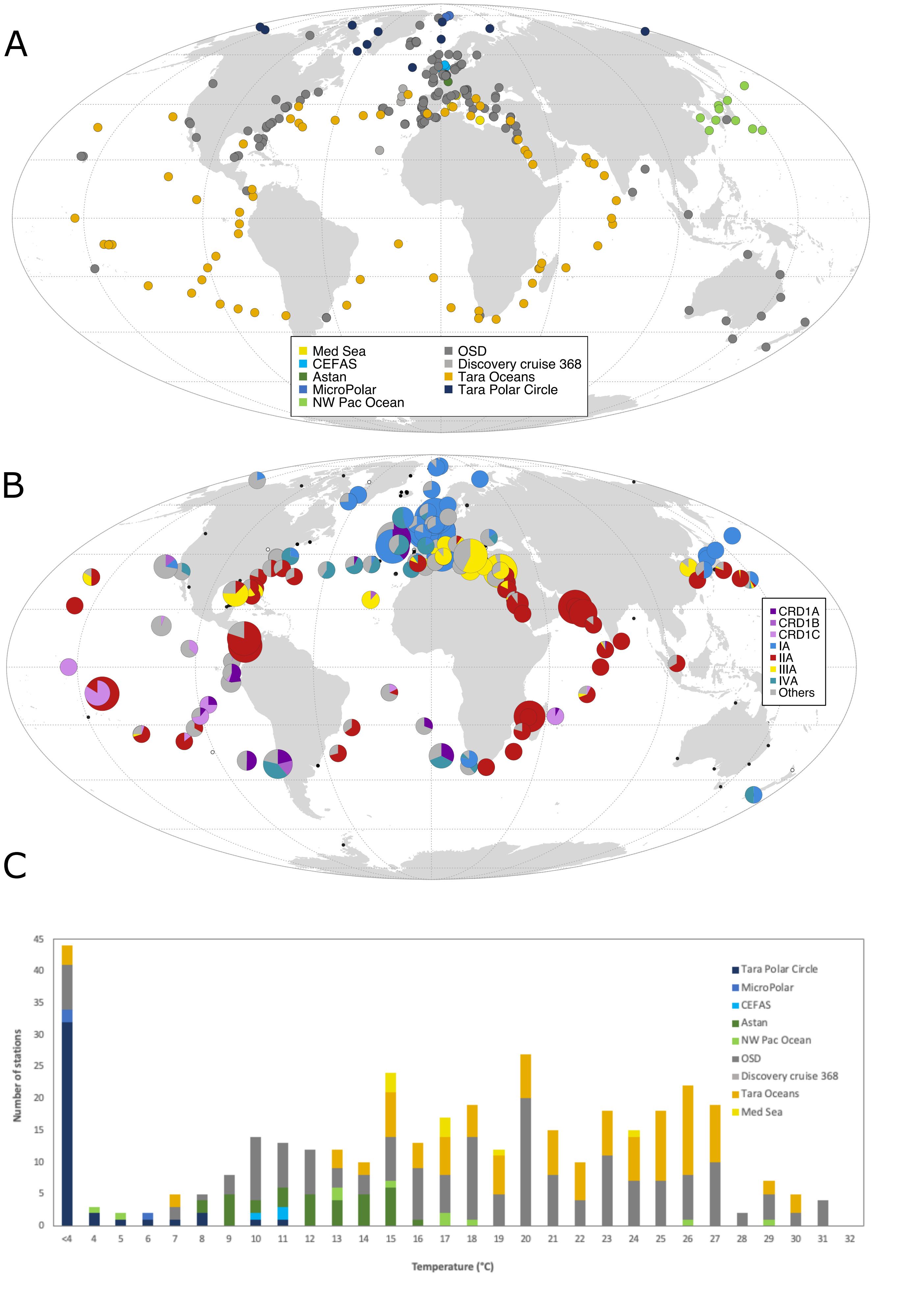


**Supplementary Figure 2. Oceanwide environmental data used in this study to determine the environmental realized thermal niches of the main ESTUs from clades CRD1 and I to IV.** **(A)** Map of the sampling sites, **(B)** Relative abundance of *Synechococcus* ESTUs IA to IVA and CRD1A to C. **(C)** Temperature distribution of the sampling sites. The inserts specify **(A,C)** the name of campaigns or datasets analyzed and **(B)** the *Synechococcus* ESTUs.

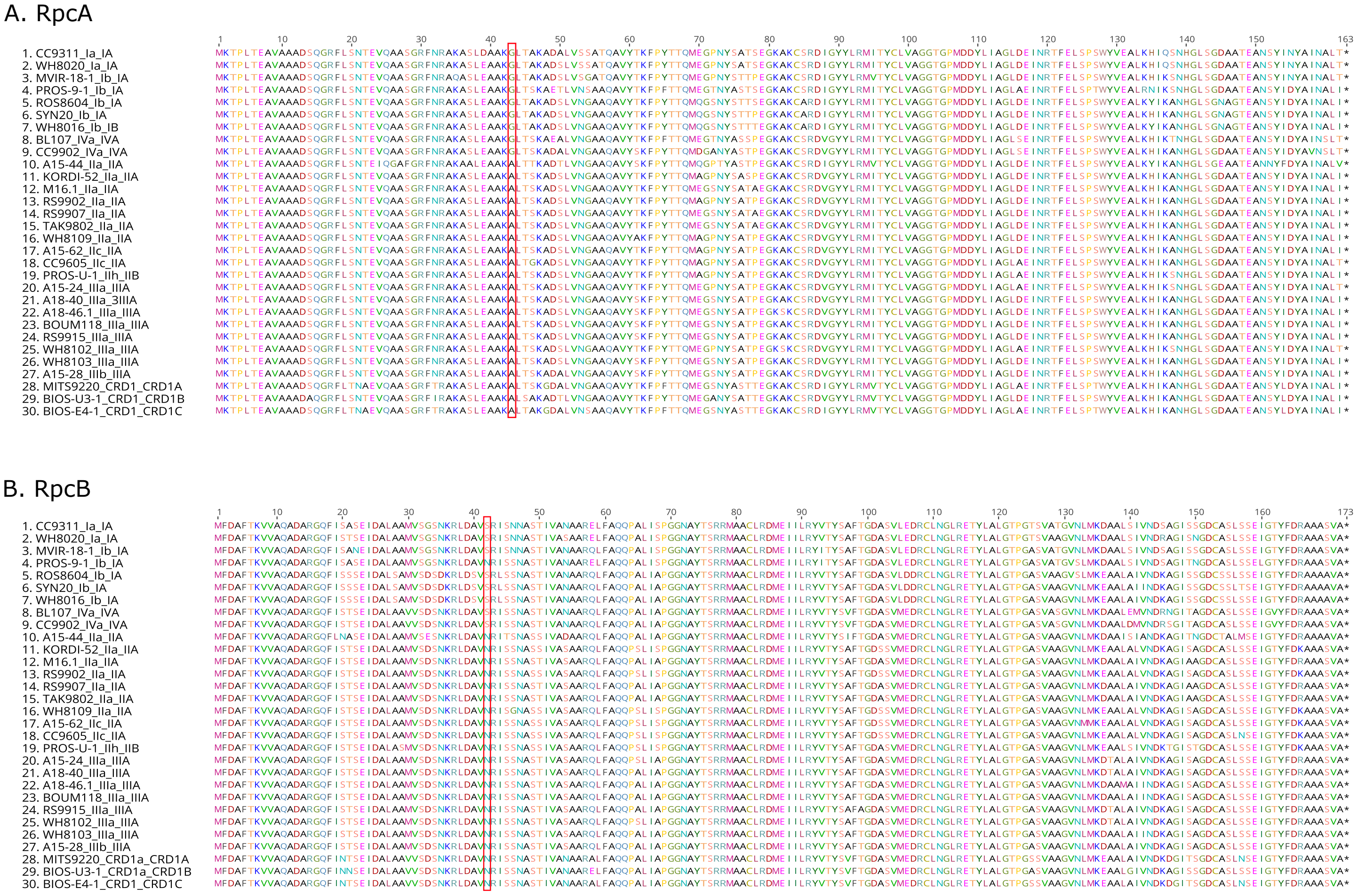


**Supplementary Figure 3. Alignment of RpcA and RpcB, encoding phycocyanin α- and β-subunits, from CRD1 and clades I-IV *Synechococcus* strains.** (A) RpcA. (B) RpcB. Substitutions potentially involved in thermotolerance are shown by a red rectangle in the alignment.

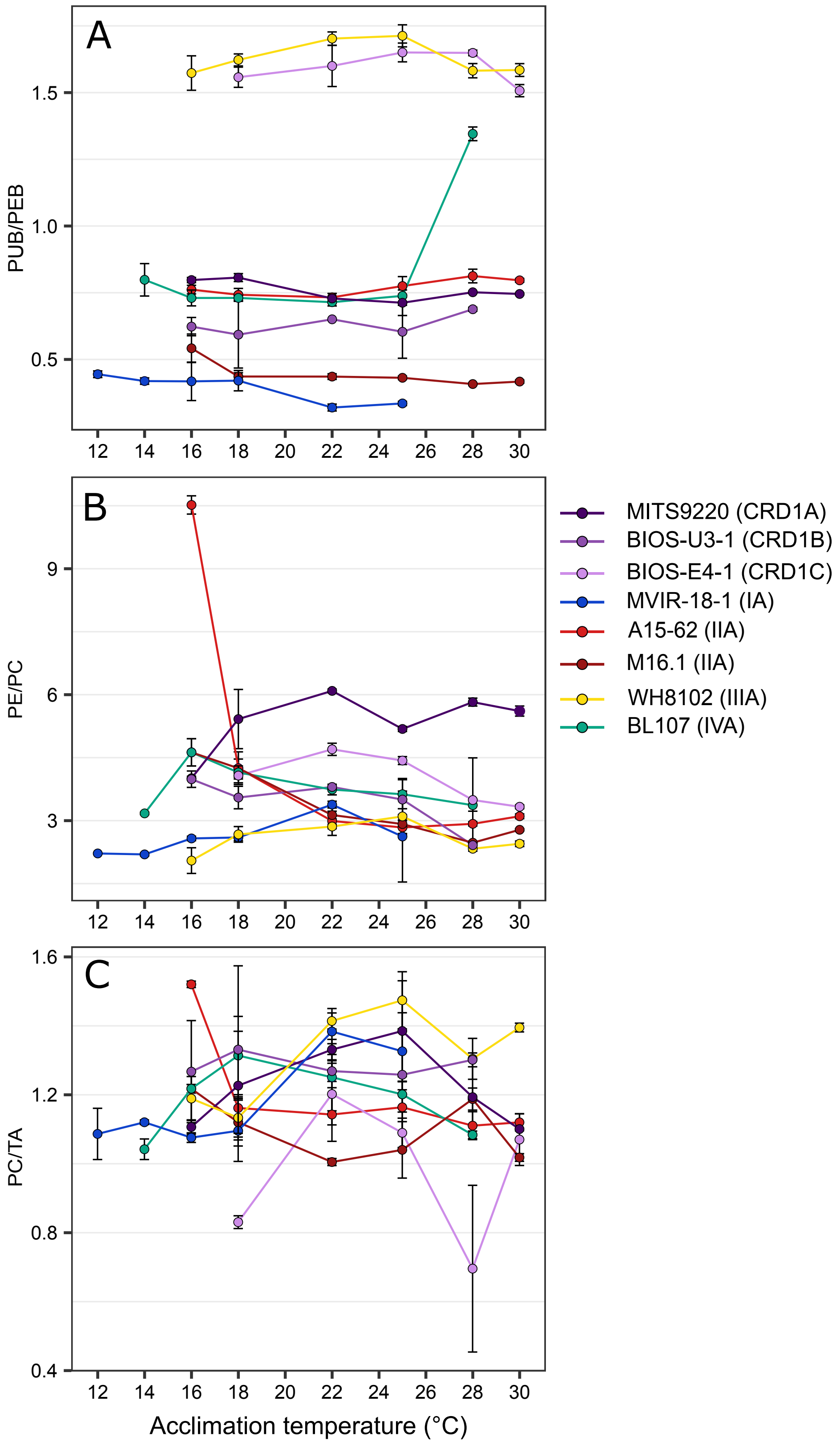


**Supplementary Figure 4. Variation with growth temperature of phycobilins and phycobiliproteins fluorescence excitation and emission ratios.** (A) Average PUB:PEB ratios. (B) Average phycoerythrin (PE) to phycocyanin (PC) ratios. (C) Average PC to terminal acceptor (TA) ratios. The insert indicates the strain names, their corresponding ESTU (sensu Farrant et al., 2016) and pigment type (sensu Humily et al., 2013) between brackets.

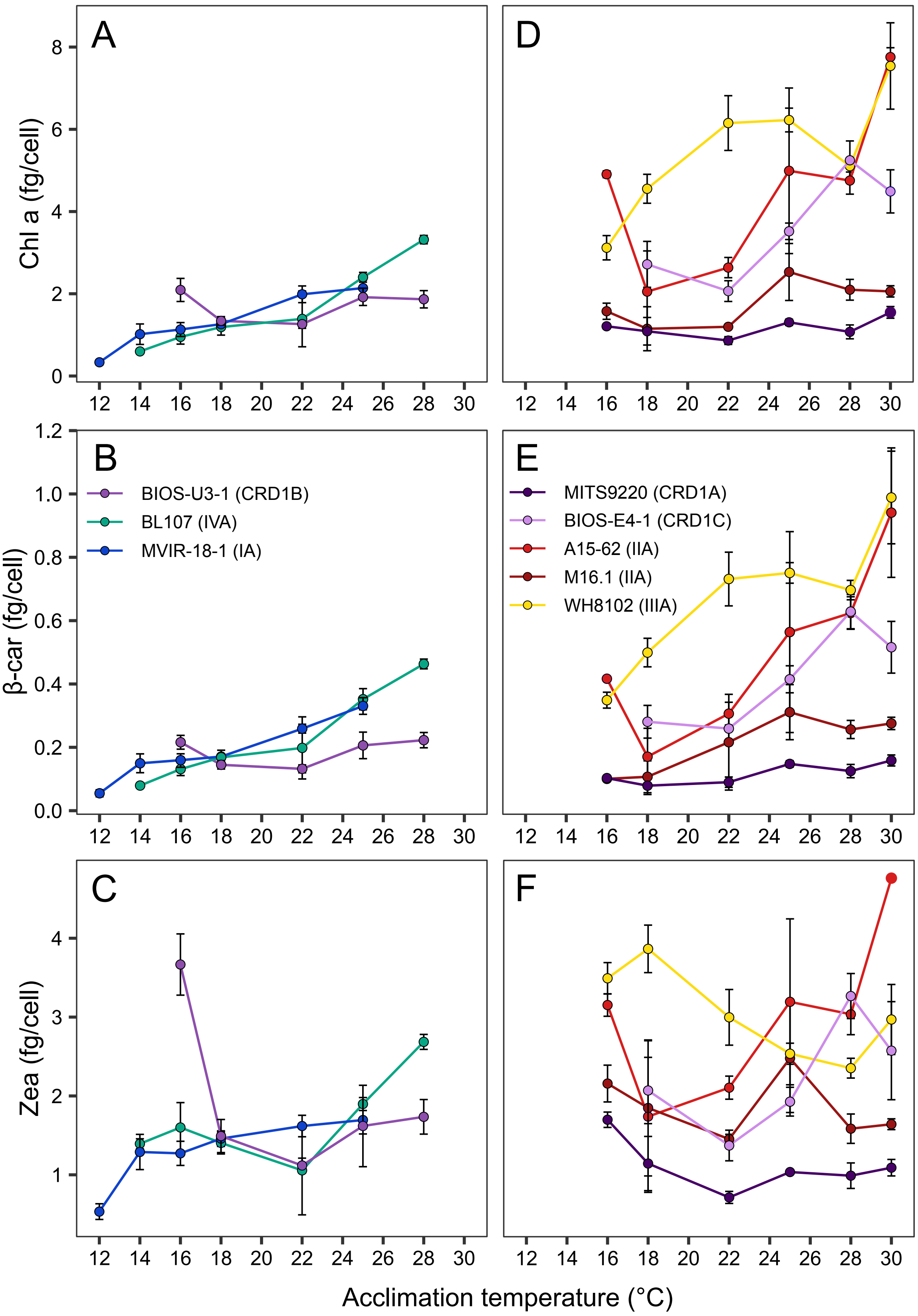


**Supplementary Figure 5. Variation with growth temperature of the three main liposoluble pigments per cell for CRD1 vs. clade I and IV strains. (A, D)** Chlorophyll (Chl) a content (fg/cell). **(B, E)** Zeaxanthin (Zea) content (fg/cell). **(C, F)** β-carotene (β-car) content (fg/cell). **(A-C)** CRD1-B strain BIOS-U3-1 vs. cold thermotypes. **(D-F)** CRD1-A strain MITS9220 and CRD1-C strain BIOS-E4-1 vs. warm thermotypes. Inserts indicate the strain names and their corresponding ESTU (*sensu* Farrant et al., 2016) between brackets.

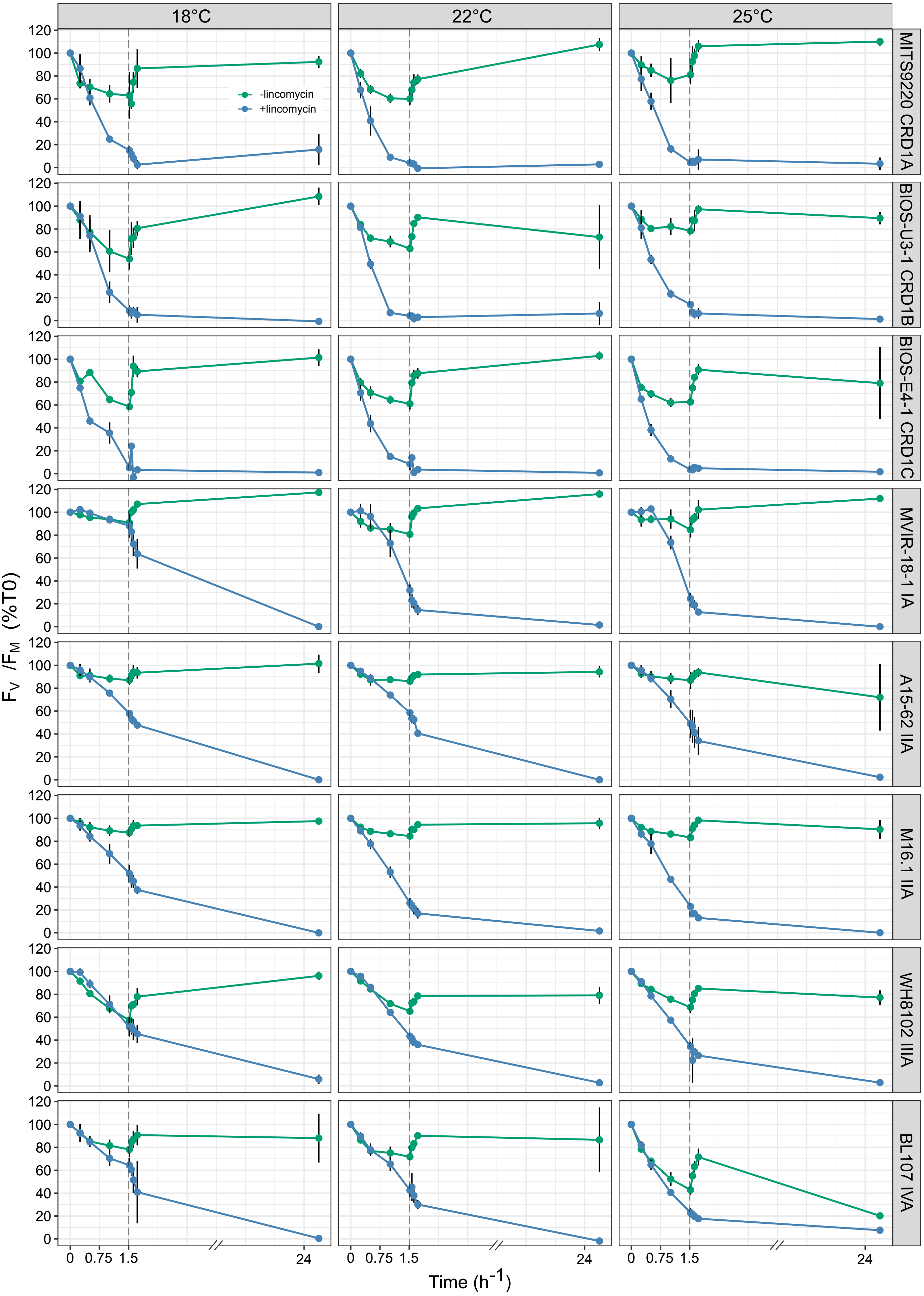


**Supplementary Figure 6. Time course of photosystem II quantum yield ($F_V/F_M$) following light stress in the presence or absence of lincomycin for CRD1 and clade I-IV strains acclimated to different temperatures.** Cultures acclimated to 75 µE m$^{-2}$ s$^{-1}$ were shifted to 375 µE m$^{-2}$ s$^{-1}$ at T0 for 90 min, then shifted back to the initial light conditions for 24h as indicated by a vertical dashed line on each figure. Strain names and their corresponding ESTU between brackets (*sensu* Farrant et al., 2016) are indicated on the right hand side, acclimation temperature are indicated on the top, whilst line colour indicates the lincomycin treatment (i.e. -/+ linco).
